# Gerofit Decreases Medication Use Among Older Veterans

**DOI:** 10.1093/geroni/igaa057.2789

**Published:** 2020-12-16

**Authors:** Orna Intrator, Jiejin Li, Peter Veazie, Sara Wesgate

**Affiliations:** 1 University of Rochester School of Medicine and Dentistry, Rochester, New York, United States; 2 Geriatrics and Extended Care Data and Analysis Center (GECDAC), Canandaigua, New York, United States; 3 VA Medical Center, Canandaigua, New York, United States

## Abstract

We examined whether Veterans enrolled in Gerofit for at least 12 months between 10/2012-3/2017 saw reductions in their medication utilization (5 sites, 226 Veterans). VA outpatient pharmacy data was used to identify medications used 12-months prior, and 12-months following Gerofit enrollment. Seven drug classes were identified (cardiovascular, diabetic, lipid, mental health, opioids, vitamins, other medications). Nearest-neighbor propensity-matched analyses was conducted with exact match on number of baseline medications and site. At baseline, Gerofit participants were taking, on average, 11.6 medications (1.7 cardiovascular, 0.6 diabetes, 0.7 lipid lowering, 0.7 mental health, 0.5 opioids, 0.6 vitamins, 7.0 other). At 12-month follow-up, Gerofit patients were taking fewer medications: any (-2.7 [-4.4, -1.0]); cardiovascular (-0.5 [-0.7, -0.3]); diabetes (-0.7 [-1.2, -0.2]); mental health (-0.9 [-1.5, -0.2]); and other (-2.4 [-3.5, -1.4]) compared to matched comparisons. No significant differences at 12-months were found for lipid lowering, opioids, or vitamins. Conclusion: Gerofit participation reduced medication use.

